# Combination of ablative fractional carbon dioxide laser and platelet-rich plasma treatment to improve hypertrophic scars: a retrospective clinical observational study

**DOI:** 10.1093/burnst/tkab016

**Published:** 2021-07-28

**Authors:** Zhanzhan Dai, Xiaozhen Lou, Tuo Shen, Yu Sun, Yongqiang Xiao, Xingfeng Zheng, Xuexin Wang, Yu Peng, Yukun Guo, Yibin Guo, Jiannan Wen, He Fang, Bing Ma, Zhaofan Xia

**Affiliations:** 1 Department of Burn Surgery, the First Affiliated Hospital of Naval Medical University; Burn Institute of PLA; 168 Changhai Road, Yangpu District, Shanghai 200433, China; 2 Research Unit of key techniques for treatment of burns and combined burns and trauma injury, Chinese Academy of Medical Sciences, 168 Changhai Road, Yangpu District, Shanghai 200433, China; 3 Department of Burn and Plastic Surgery, the 970th Hospital of People's Liberation Army, 7 Zhichunan Road, Zhifu District, Yantai, Shandong, 264000, China; 4 Department of Health Statistics, the Naval Medical University, 800 Xiangyin Road, Yangpu District, Shanghai 200433, China; 5 First Resident Outpatient Department of Northern Theater General Hospital, 22 Beiwu Road, Heping District, Shenyang, Liaoning Province, 110001, China

**Keywords:** Ablative fractional carbon dioxide laser, Platelet-rich plasma, Hypertrophic scars

## Abstract

**Background:**

Hypertrophic scars are one of the main complications that affect the quality of life of patients after burns. Many methods have been shown to be effective in the treatment of hypertrophic scars, such as ablative fractional CO_2_ laser (AFCL) and platelet-rich plasma (PRP). However, there are few studies on the effect of the combined application of these measures. The purpose of this study was to explore the therapeutic effect of AFCL combined with PRP on hypertrophic burn scars.

**Methods:**

A retrospective clinical observation study was conducted on 50 patients with hypertrophic burn scars. The AFCL+PRP group included 31 patients who received AFCL combined with PRP treatment; the AFCL group included 19 patients who received AFCL treatment only. The University of North Carolina 4P Scar Scale (UNC4P) and the Vancouver Scar Scale (VSS) scores that were collected before each treatment were used as indicators of the effectiveness of the previous treatment. The scores recorded at the second, fourth and seventh months were analysed.

**Results:**

The demographic data of the 2 groups were not significantly different. Before treatment, there was no difference in the UNC4P and VSS scores between the 2 groups. There was a significant decline in the UNC4P and VSS total scores over 6 months in both groups (*p* < 0.05) and scores in the 2 groups were comparable after 3 and 6 months (*p* < 0.05). UNC4P scores in the AFCL+PRP group decreased from a mean of 8.26 to 2.61 (*p* < 0.05) with a concomitant drop in VSS scores from a mean of 11.74 to 6.06 (*p* < 0.01). In the AFCL group UNC4P and VSS scores decreased from 7.68 to 4.63 (*p* < 0.05) and from 10.89 to 8.16 (*p* < 0.05), respectively. The sub-items of these 2 assessments were analysed and the results suggest that AFCL combined with PRP can comprehensively improve scarring.

**Conclusions:**

This study shows that PRP is an effective adjunct for AFCL in the treatment of hypertrophic burn scars and that the combination of PRP and AFCL proved to be more useful than AFCL alone. This combination may be a new and effective clinical practice for the treatment of scars. However, larger and higher-level clinical studies are still needed to determine its efficacy and possible mechanisms.

Highlights

- We explored the therapeutic effect of ablative fractional CO_2_ laser (AFCL) combined with platelet-rich plasma (PRP) on hypertrophic scars.
- Our results suggest that AFCL combined with PRP can comprehensively improve hypertrophic scars.
- The combination of PRP and AFCL proved more useful than AFCL alone.

## Background

Hypertrophic scars are the main clinical result of full-thickness burn wounds. Their prevalence has been reported to be as high as 70% [1], independent of treatment strategy. The disfigurement, pain, itching and contracture deformity caused by hypertrophic scars place a large psychological burden on patients and affect their quality of life [2]. The mechanism of scar formation is relatively well understood: it is mainly caused by the massive increase in collagen and fibre dimensions during the repair of the damaged area [3]. Although there are many methods used in scar treatment, such as physiotherapy, compression, topical drugs and surgery, these methods still have some limitations [4]. Since Hantash *et al.* [5] first applied ablative fractional CO_2_ laser (AFCL) in the treatment of hypertrophic burn scars in 2007, its therapeutic effects have become increasingly evident [6]. Platelet-rich plasma (PRP) has been proven useful in many fields, especially because it releases high concentrations of growth factors and enhances cell proliferation and angiogenesis to promote tissue repair and reduce scar formation [7]. Several studies have investigated the effect of PRP and AFCL on acne scarring. However, there are few studies on the effect of PRP and AFCL on hypertrophic burn scars. Therefore, we conducted the present study to explore the effects of PRP combined with AFCL in patients with hypertrophic scars.

## Methods

### Study setting

This retrospective controlled study met the basic requirements of the Helsinki Declaration. According to the policy of the Ethics Committee of the First Affiliated Hospital of Naval Medical University, clinical data can be analysed and used without revealing the identity of the patient.

Ethical approval and consent were obtained from Shanghai Changhai Hospital Ethics Committee (CHEC2014-096) for the data in this research. And the patient’s clinical data can be used for research purposes.

A retrospective and comparative clinical observation study was conducted on 50 burn patients with hypertrophic scars who attended the burn centre at the hospital affiliated to the Naval Medical University in China during the period from August 2017 to March 2020. Among the enrolled patients, 31 patients (19 men and 12 women), with a mean age of 41 years (range, 22–75), were treated with AFCL and PRP combined; we termed these the AFCL+PRP group. The remaining 19 patients (8 men and 11 women), with a mean age of 40 years (range 20–59), underwent AFCL only. Eligible patients had to meet the following inclusion criteria: (1) diagnosed with hypertrophic burn scar; (2) aged >16 years; (3) completed a total of 7 sessions of AFCL or AFLC+PRP treatments once per month within 6 months; and (4) have complete follow-up and clinical information record. The exclusion criteria were as follows: (1) infection in the scar area; (2) pregnant or lactating women; (3) use of drugs or related blood diseases that affect platelet concentration or function; (4) retinoid therapy within 6 months of the beginning of the study; and (5) chronic wound or scar rupture.

### Preparation of PRP

The 2-step centrifugation method was used in this case series as follows. Before the treatment, 10 ml of venous blood was drawn from the patient’s peripheral access into a blood collection tube containing sodium citrate as an anticoagulant and transferred to a centrifuge (PRP preparation kits, Shandong Weigao Group Medical Polymer Company Ltd, China). After centrifugation at 2750 revolutions per minute for 10 minutes, the blood sample was separated into 3 layers. Using a needle, the lowest layer, containing red blood cells, was extracted and the remaining parts were centrifuged again at 2750 revolutions per minute for 5 minutes. After removing the upper layer, PRP was separated out.

**Table 1:** Demographic characteristics of hypertrophic scar patients

**Variables**	**AFCL (n = 19)**	**AFCL+PRP (n = 31)**	**Statistical variables**	***P* value**
Male, n(%)	8 (42.1%)	19 (61.3%)	*χ* ^2^ = 1.746	0.186
Age, years (mean ± SD)	40 ± 10.31	41 ± 12.70	*t* = 0.409	0.685
BMI	23.08 ± 2.51	22.84 ± 3.01	*t* = 0.279	0.781
TBSA of burn, % (mean ± SD)	48.00 ± 26.49	37.58 ± 28.24	*t* = 1.290	0.203
Skin graft, n(%)	10 (52.6%)	15 (48.4%)	*χ* ^2^ = 0.005	0.944

*AFCL* ablative fractional CO_2_ laser, *PRP* platelet-rich plasma, *BMI* body mass index, *TBSA* total body surface area

### Treatment protocol

Different anaesthetic methods were applied according to the size of the scar: general anaesthesia or spinal anaesthesia for large scars (>20% total body surface area (TBSA)) and local anaesthesia with compound lidocaine cream for smaller scars (≤20% TBSA). After anaesthesia, the treatment area was sterilized with chlorhexidine before each treatment procedure. The patients underwent 10,600 nm fractional CO_2_ laser exposures (Ultralise Encore, Lumennis Co. Ltd, China) for the whole scar, using the deep tissue handpiece with an energy of 17.5–100 mJ, a density of 3–5% and a frequency of 250 Hz. Wound care after laser therapy included application of Prontosan gel (B. Braun Medical AG, Germany) and changing the external dressing 5–10 days after each AFCL operation. For the AFCL+PRP group, the PRP was spread evenly onto the wounds following AFCL operations. Appropriate wound dressings were used to cover the treated areas for 7 days following laser treatment. Sessions were conducted every month for 6 consecutive sessions.

### Data collection and outcome measures

All cases were evaluated by 2 blinded dermatologists using the University of North Carolina 4P Scar Scale (UNC4P) and the Vancouver Scar Scale (VSS) before every session and 1 month after the last session. The UNC4P scale includes pliability, pruritus, pain and paraesthesia. The VSS includes pigmentation, vascularity, height and pliability. Assessment was documented before every session and 1 month after the final session.

### Statistical analysis

The collected data were revised, tabulated and analysed using IBM SPSS version 24 software (IBM SPSS, Chicago, IL, USA). The quantitative variables are presented as the mean ± standard deviation and the categorical variables as frequencies (and proportions). Student’s *t*-test and the chi-square test were used to compare the patient demographics. Two-way repeated measures analysis of variance and the Mann–Whitney *U* test were used to compare the scores of 2 groups. All statistical tests were two-sided and *p* values <0.05 were considered statistically significant.

## Results

### Patient demographics

A total of 50 patients were retrospectively studied, of whom 31 were in the AFCL+PRP group and 19 in the AFCL group. There were no significant differences between the intraoperative analgesic regimens with respect to sex, age, body mass index or TBSA of burn between the 2 groups (Table 1).

### Comparing the improvement between AFCL and AFCL+PRP groups

Before treatment, the UNC4P (7.68 ± 1.17 *vs* 8.26 ± 1.22) and VSS (10.89 ± 1.65 *vs* 11.74 ± 1.61) scores were not significantly different between the AFCL and AFCL+PRP groups. The sub-items of both scales also showed no statistically significant differences (Table 2).

**Table 2:** Improvement of UNC4P and VSS scores of hypertrophic scar patients after AFCL and AFCL+PRP treatments

	**AFCL**				**AFCL+PRP**			
	**Pre-treatment**	**First month**	**Third month**	**Sixth month**	**Pre-treatment**	**First month**	**Third month**	**Sixth month**
UNC4P	7.68 ± 1.17	6.84 ± 1.27^a^	5.95 ± 0.55^a^	4.63 ± 1.09^a^	8.26 ± 1.22	6.52 ± 1.16^b^	4.68 ± 1.28^b^^,^^c^	2.61 ± 1.29^b^^,^^c^
Pliability	1.89 ± 0.31	1.89 ± 0.31	1.89 ± 0.31	1.68 ± 0.46^a^	2.00 ± 0.25	2.00 ± 0.25	1.90 ± 0.30	1.19 ± 0.40^b^^,^^c^
Pruritus	2.00 ± 0.46	1.58 ± 0.59^a^	1.05 ± 0.51^a^	0.74 ± 0.44^a^	2.13 ± 0.55	1.23 ± 0.49^b^^,^^c^	0.55 ± 0.56^b^^,^^c^	0.16 ± 0.37^b^^,^^c^
Pain	1.95 ± 0.60	1.53 ± 0.60^a^	1.21 ± 0.41^a^	1.00 ± 0.32^a^	2.06 ± 0.62	1.45 ± 0.50	0.90 ± 0.47^b^^,^^c^	0.45 ± 0.50^b^^,^^c^
Paraesthesia	1.84 ± 0.49	1.84 ± 0.49	1.74 ± 0.55	1.21 ± 0.61^a^	2.06 ± 0.44	1.84 ± 0.51	1.32 ± 0.47^b^^,^^c^	0.80 ± 0.59^b^^,^^c^
VSS	10.89 ± 1.65	10.68 ± 1.66	9.89 ± 1.62^a^	8.16 ± 1.93^a^	11.74 ± 1.61	10.39 ± 1.31	8.45 ± 1.04^b^^,^^c^	6.06 ± 1.44^b^^,^^c^
Pigmentation	2.89 ± 0.45	3.00 ± 0	3.00 ± 0	2.68 ± 0.73	3.00 ± 0	3.00 ± 0	2.87 ± 0.49	2.16 ± 0.99^b^
Vascularity	3.21 ± 0.77	2.89 ± 0.97	2.37 ± 0.98^a^	1.79 ± 0.69^a^	3.61 ± 0.94	2.58 ± 0.84^b^	1.77 ± 0.61^b^^,^^c^	1.06 ± 0.35^b^^,^^c^
Height	2.00 ± 0.32	2.10 ± 0.45	2.16 ± 0.49	2.11 ± 0.55	2.19 ± 0.47	2.23 ± 0.49	1.87 ± 0.49^b^	1.68 ± 0.47^b^^,^^c^
Pliability	2.79 ± 0.61	2.68 ± 0.65	2.37 ± 0.48^a^	1.58 ± 0.59^a^	2.94 ± 0.50	2.58 ± 0.55^b^	1.94 ± 0.35^b^^,^^c^	1.16 ± 0.37^b^^,^^c^

*UNC4P* University of North Carolina 4P Scar Scale, *VSS* Vancouver Scar Scale, *AFCL* ablative fractional CO_2_ laser, *PRP* platelet-rich plasma

^a^ *p* < 0.05 compared with pre-treatment value in AFCL group

^b^ *p* < 0.05 compared with pre-treatment value in AFCL+PRP group

^c^ *p* < 0.05 compared with the value of the AFCL group at the same time point

**Figure 1.**
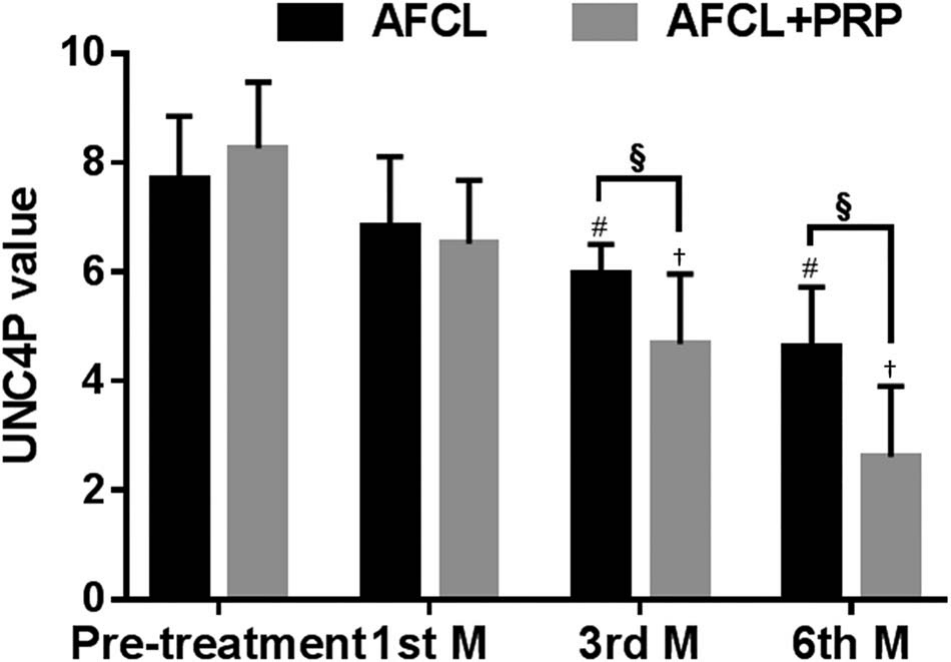
UNC4P values after AFCL and AFCL+PRP treatment. ^#^*p* < 0.05 compared with pre-treatment value in the AFCL group; ^†^*p* < 0.05 compared with pre-treatment value in the AFCL+PRP group; ^§^*p* < 0.05 compared with the value of the AFCL group at the same time point. *UNC4P* University of North Carolina 4P Scar Scale, *AFCL* ablative fractional CO_2_ laser, *PRP* platelet-rich plasma, *M*, month

**Figure 2.**
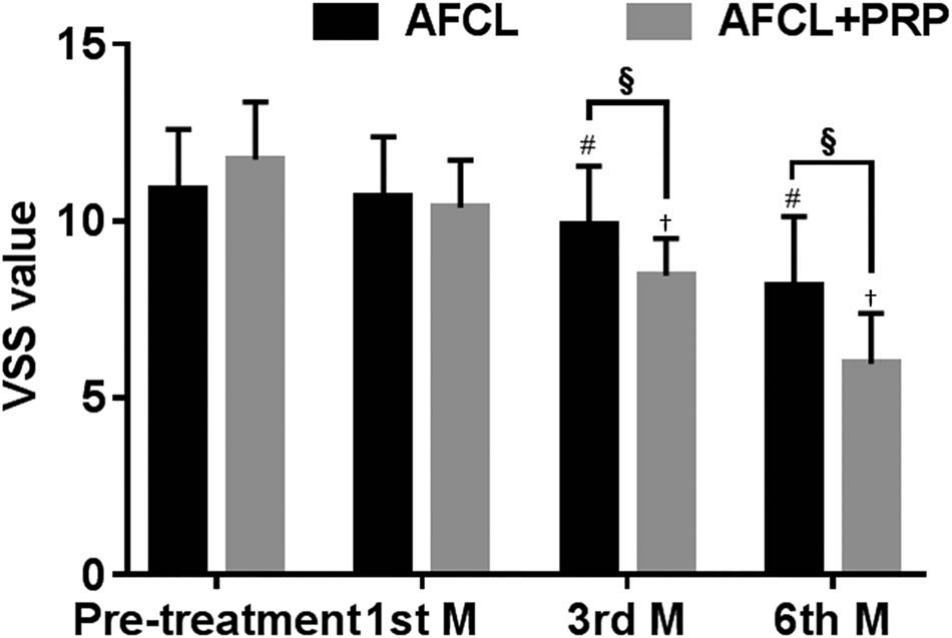
VSS values after AFCL and AFCL+PRP treatment. ^#^*p* < 0.05 compared with pre-treatment value in the AFCL group; ^†^*p* < 0.05 compared with pre-treatment value in the AFCL+PRP group; ^§^*p* < 0.05 compared with the value of the AFCL group at the same time point. *VSS* Vancouver Scar Scale, *AFCL* ablative fractional CO_2_ laser, *PRP* platelet-rich plasma, *M*, month

In the AFCL group, the scores from both scales decreased after treatment compared with before treatment. The UNC4P scores after 1, 3 and 6 months were 6.84 ± 1.27, 5.95 ± 0.55 and 4.63 ± 1.09, respectively; the scores after 3 and 6 months were significantly lower than those before treatment (*p* < 0.05; Figure 1). The VSS scores after 1, 3 and 6 months were 10.68 ± 1.66, 9.89 ± 1.62 and 8.16 ± 1.93, respectively; the scores after 3 and 6 months were significantly lower than those before treatment (*p* < 0.05; Figure 2). This suggests that AFCL plays a positive role in improving scar formation.

In the AFCL+PRP group, the UNC4P and VSS scores improved similarly. After 1, 3 and 6 months of treatment, the UNC4P scores were 6.52 ± 1.16, 4.68 ± 1.28 and 2.61 ± 1.29, respectively; the scores after 3 and 6 months were significantly lower than those before treatment (*p* < 0.05; Figure 1). The VSS scores were 10.39 ± 1.31, 8.45 ± 1.04 and 6.06 ± 1.44, respectively, after 1, 3 and 6 months of treatment; the scores after 3 and 6 months were significantly lower than those before treatment (*p* < 0.05; Figure 2).

After combined treatment with AFCL and PRP, the UNC4P and VSS scores were significantly lower than those after AFCL treatment alone. The differences between the UNC4P and VSS scores at the third and sixth months were statistically significant (*p* < 0.05). This suggests that the combined application of AFCL+PRP can improve scars more effectively.

**Figure 3.**
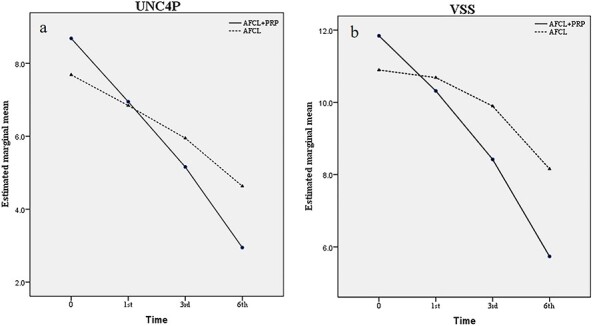
Estimated marginal mean UNC4P (a) and VSS (b) scores by time. The decrease of UNC4P (a) and VSS (b) scores were observed in different treatment groups; however, the UNC4P scores in AFCL+PRP group showed a more obvious downward trend than that in AFCL group (a), and the different downward trend was statistically significant (difference, −1.68; 95% confidence interval (CI), −2.47, −0.80; *p* < 0.001). The VSS score showed a similar downward trend in AFCL+PRP group and AFCL group (difference, −2.42; 95% CI, −3.68, −1.16; *p* < 0.001). *UNC4P* University of North Carolina 4P Scar Scale, *VSS* Vancouver Scar Scale

**Figure 4.**
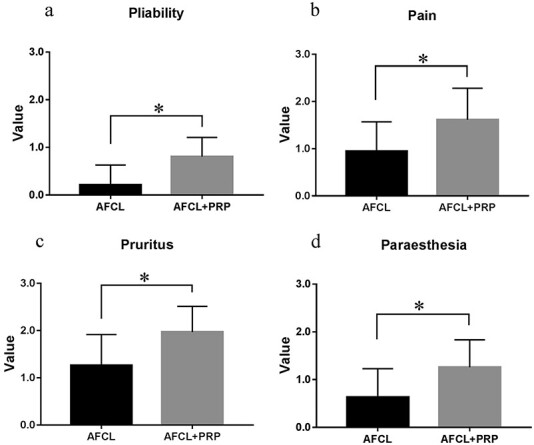
Improvements of pliability (a), pain (b), pruritus (c) and paresthesia (d) as scored by the UNC4P before and after AFCL and AFCL+PRP treatment. After 6 months treatment, the pliability (a), pain (b), pruritus (c) and paresthesia (d) scores on the UNC4P in the AFCL+PRP group were reduced by 0.81 ± 0.07, 1.61 ± 0.12, 1.97 ± 0.10 and 1.26 ± 0.10, respectively, resulting in significantly lower scores than that in the AFCL group (0.21 ± 0.10, 0.95 ± 0.14, 1.26 ± 0.15 and 0.63 ± 0.14, respectively; *p* < 0.05). ^*^p < 0.05, MannWhitney U test. *UNC4P* University of North Carolina 4P Scar Scale, *AFCL* ablative fractional CO_2_ laser, *PRP* platelet-rich plasma

**Figure 5.**
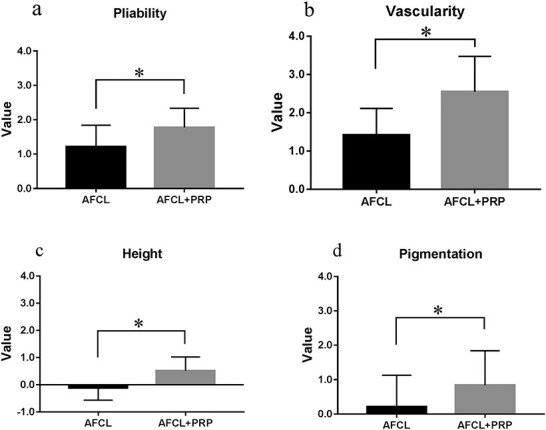
Improvement of pliability (a), vascularity (b), height (c) and pigmentation (d) as scored by the VSS before and after AFCL and AFCL+PRP treatment. After 6 months treatment, the pliability (a), vascularity (b), height (c) and pigmentation (d) scores on the VSS in the AFCL+PRP group decreased by 1.77 ± 0.10, 2.55 ± 0.17, 0.52 ± 0.09 and 0.84 ± 0.18, respectively, resulting in significantly lower scores than in the AFCL group (1.21 ± 0.14, 1.42 ± 0.16, −0.11 ± 0.11 and 0.21 ± 0.21, respectively; *p* < 0.05). ^*^p < 0.05, MannWhitney U test. *VSS* Vancouver Scar Scale, *AFCL* ablative fractional CO_2_ laser, *PRP* platelet-rich plasma

The marginal mean estimation method was adopted to analyse the influence of different treatment methods on scar formation. Repeated measures analysis of variance was used to analyse the influence of different treatment methods on scar formation. As mentioned above, the UNC4P scores showed a downward trend after both treatments, and the downward trend in the AFCL+PRP group was more obvious than that in the AFCL group (difference, −1.68; 95% confidence interval (CI), −2.47, −0.80; *p* < 0.001; Figure 3a). The VSS scores showed a similar downward trend (difference, −2.42; 95% CI, −3.68, −1.16; *p* < 0.001; Figure 3b).

From the results in Table 1, it can be seen that after treatment with AFCL and AFCL+PRP, the UNC4P and VSS scores were lower than before treatment, which means that the patients’ scars had improved. In order to analyse in detail the effect of these 2 treatments on scars, the sub-items of the 2 assessments were analysed. The score at 6 months of treatment minus the score before treatment was used to measure the effect of different treatment methods on the UCN4P and VSS sub-items.

After 6 months, the pliability, pain, pruritus and paraesthesia scores on the UNC4P in the AFCL group decreased by 0.21 ± 0.10, 0.95 ± 0.14, 1.26 ± 0.15 and 0.63 ± 0.14, respectively. The same scores on the UNC4P in the AFCL+PRP group were reduced by 0.81 ± 0.07, 1.61 ± 0.12, 1.97 ± 0.10 and 1.26 ± 0.10, respectively, resulting in significantly lower scores than in the AFCL group (*p* < 0.05; Figure 4). During the same period, the pliability, vascularity, height and pigmentation scores on the VSS in the AFCL group decreased by 1.21 ± 0.14, 1.42 ± 0.16, −0.11 ± 0.11 and 0.21 ± 0.21, respectively. The same scores on the VSS in the AFCL+PRP group were reduced by 1.77 ± 0.10, 2.55 ± 0.17, 0.52 ± 0.09 and 0.84 ± 0.18, respectively, resulting in significantly lower scores than in the AFCL group (*p* < 0.05; Figure 5). These results suggest that AFCL combined with PRP could comprehensively improve scar formation.

## Discussion

There have been many reports on the application of PRP and AFCL in the treatment of scars, but most of these studies were on acne scars [8–10]. The present study focused on the effect on hypertrophic scarring following a burn-related injury. The results show that the combination of PRP and AFCL significantly improved the quality of scars and exerted a superior effect compared with the use of PRP alone.

PRP is a new treatment—a plasma therapy with a high concentration of platelets, containing vascular endothelial growth factor, transforming growth factor, epidermal growth factor, fibroblast growth factor and so forth. It provides raw materials for tissue repair while recruiting stem cells to the local area of the skin lesions and promoting their proliferation to achieve the goals of skin tissue regeneration and control of excessive inflammatory reactions during the process of early wound repair [11]. The validity of laser therapy in the treatment of hypertrophic scarring was verified again in our study, as it previously has been in the literature [12,13]. Hultman *et al.* also reported a significant decrease in the UNC4P and VSS scores of 147 burn patients after a single laser treatment [6]. Therefore, we chose these 2 scales to measure the effect. A recent study [14] has shown that high-density fractional CO_2_ laser treatment provides more improvement in burn scars both clinically and histopathologically, especially in the parameters of pliability and relief. There are also many reports of the effectiveness of other treatment methods combined with PRP to treat hypertrophic> burn scars, such as autologous fat injection [15] and narrow-spectrum intense pulsed light [16].

Although the VSS is a classic and commonly used scale, it still has some shortcomings. In our study, the pigmentation of scars improved significantly after treatment in both groups, but it nonetheless appeared as pigmentation. Because the VSS score for pigmentation only contains 3 grades, improvement of pigmentation cannot be reflected in this scale. When evaluating the pigmentation of scars, researchers can make some improvements to the VSS or use other assessment scales to compensate for this deficiency.

A recent study by Neinaa *et al.* [17] reported significant clinical improvement of post-acne scars treated with AFCL combined with PRP, as evidenced by the significant reduction in both the Clinique des Cicatrices d’Acné and Goodman and Baron’s qualitative scar scale scores with minimal adverse effects. In that study, they found that AFCL-assisted delivery of lyophilized growth factors, a new PRP preparation that is standardized in growth factor concentrations, reached a higher degree of clinical improvement and patient satisfaction. This is a huge technological advance that has great prospects for development.

Fractional laser therapy creates precise, uniform columns of tissue vaporization, which helps to facilitate drug delivery past the epidermal barrier and even distribution of drugs in the dermal layer [18].

Some deficiencies exist in our research, such as the lack of an objective evaluation methodology, statistics, analysis of complications and a histopathological assessment. Other researchers have used different direct measurements of lesions, including Acosta *et al.* [19], who incorporated the dimensions of the lesions observed with sonography, and Weshahy [20], who used an alginate mould filled with saline solution to judge the volume more accurately.

## Conclusions

The present study showed that PRP is an effective adjunct for AFCL in the treatment of hypertrophic burn scars, and the combination of PRP and AFCL proved to be more useful than AFCL alone. This combination may be a new and effective clinical practice for the treatment of scars. However, larger and higher-level clinical studies are still needed to determine its efficacy and possible mechanisms.

## Authors’ contributions

ZZD, XZL and TS were responsible for writing the manuscript and producing the figures. YS, YQX and XFZ were responsible for searching the literature. XXW, YP, YKG, YBG and JNW were responsible for data interpretation, data analysis and editing the manuscript. HF, BM and ZFX were responsible for the research design, review and quality supervision of the manuscript.

## Funding

This paper was supported by the National Nature Science Foundation of China (81701899), the Youth Incubation Plan of the Military Medical Science and Technology (16QNP091), the CAMS Innovation Fund for Medical Sciences (2019-I2M-5-076) and the high level achievement cultivation plan of the Naval Medical University (2018-CGPZ-B03).

## Conflicts of interest

The authors declare that they have no competing interests.

## Ethics approval and consent to participate

This study was approved by the Shanghai Changhai Hospital Ethics Committee (CHEC2014-096) and the consent of clinical data for research purposes was obtained from the patients.

## Abbreviations

AFCL: ablative fractional CO_2_ laser; PRP: platelet-rich plasma; TBSA: total body surface area; UNC4P: University of North Carolina 4P Scar Scale; VSS: Vancouver Scar Scale.

## References

[1] Bombaro KM , Engrav LH, Carrougher GJ, Wiechman SA, Faucher L, Costa BA, et al. What is the prevalence of hypertrophic scarring following burns? Burns. 2003;29:299–302. 12781605 10.1016/s0305-4179(03)00067-6

[2] Monsuur HN , van den Broek LJ, Jhingoerie RL, Vloemans AFPM, Gibbs S. Burn eschar stimulates fibroblast and adipose mesenchymal stromal cell proliferation and migration but inhibits endothelial cell sprouting. Int J Mol Sci. 2017;18(8):1790. doi:10.3390/ijms18081790. PMC5578178 28820426

[3] Gold MH , McGuire M, Mustoe TA, Pusic A, Sachdev M, Waibel J, et al. Updated international clinical recommendations on scar management: part 2--algorithms for scar prevention and treatment. Dermatol Surg. 2014;40:825–31. 25068544 10.1111/dsu.0000000000000050

[4] Anderson RR , Donelan MB, Hivnor C, Greeson E, Ross EV, Shumaker PR, et al. Laser treatment of traumatic scars with an emphasis on ablative fractional laser resurfacing: consensus report. JAMA Dermatol. 2014;150:187–93. 24336931 10.1001/jamadermatol.2013.7761

[5] Hantash BM , Bedi VP, Chan KF, Zachary CB. Ex vivo histological characterization of a novel ablative fractional resurfacing device. Lasers Surg Med. 2007;39:87–95. 17115384 10.1002/lsm.20405

[6] Hultman CS , Friedstat JS, Edkins RE, Cairns BA, Meyer AA. Laser resurfacing and remodeling of hypertrophic burn scars: the results of a large, prospective, before-after cohort study, with long-term followup. Ann Surg. 2014;260:519–29. 25115428 10.1097/SLA.0000000000000893

[7] Mazzucco L , Borzini P, Gope R. Platelet-derived factors involved in tissue repair-from signal to function. Transfus Med Rev. 2010;24:218–34. 20656189 10.1016/j.tmrv.2010.03.004

[8] Wu N , Sun H, Sun Q, Cong L, Liu C, Zheng Y, et al. A meta-analysis of fractional CO2 laser combined with PRP in the treatment of acne scar. Lasers Med Sci. 2021. https://doi.org/10.1007/s10103-020-03105-z. 32827074

[9] Al Taweel AI , Al Refae AA, Hamed AM, Kamal AM. Comparative study of the efficacy of platelet-rich plasma combined with carboxytherapy vs its use with fractional carbon dioxide laser in atrophic acne scars. J Cosmet Dermatol. 2019;18:150–5. 29682870 10.1111/jocd.12561

[10] Abdel Al AM , Ibrahim IM, Sami NA, Abdel Kareem IM. Evaluation of autologous platelet-rich plasma plus ablative carbon dioxide fractional laser in the treatment of acne scars. J Cosmet Laser Ther. 2018;20:106–13. 28853968 10.1080/14764172.2017.1368667

[11] Hesseler MJ , Shyam N. Platelet-rich plasma and its utility in medical dermatology: a systematic review. J Am Acad Dermatol. 2019;81:834–46. 31009668 10.1016/j.jaad.2019.04.037

[12] Hultman CS , Edkins RE, Wu C, Calvert CT, Cairns BA. Prospective, before-after cohort study to assess the efficacy of laser therapy on hypertrophic burn scars. Ann Plast Surg. 2013;70:521–6. 23542846 10.1097/SAP.0b013e31827eac5e

[13] Xie WG , Lei F, Wang J, Xu J, Ruan JJ, Li Z. Clinical effects of sequential laser treatments on early stage hypertrophic burn scars. Chin J Burns. 2018;34:615–23. 10.3760/cma.j.issn.1009-2587.2018.09.011 30293365

[14] Tawfic S , Sayed S, Nada A, Manaa D, Shalaby S. High- versus low-density fractional laser in the treatment of hypertrophic postburn scars: a randomized clinical trial. Dermatol Surg. 2020;46:e38–44. 31851017 10.1097/DSS.0000000000002293

[15] Huang Z , Chen Y, Wang P, Zheng DW, Zong YL, Lyu GZ. A prospective randomized controlled clinical study on the treatment of hypertrophic scar after burn by fractional carbon dioxide laser combined with autologous fat injection. Chin J Burns. 2021;37:49–56. 10.3760/cma.j.cn501120-20200104-00002 33499569

[16] Li N , Yang L, Cheng J, Han JT, Hu DH. Clinical comparative study of pulsed dye laser and ultra-pulsed fractional carbon dioxide laser in the treatment of hypertrophic scars after burns. Chin J Burns. 2018;34:603–7. 10.3760/cma.j.issn.1009-2587.2018.09.009 30293363

[17] Neinaa YME , Al-Khayat LA, Suliman GAM, Ameen TA. Fractional carbon dioxide laser assisted delivery of lyophilized-growth factors is a promising treatment modality of post-acne scars. Dermatol Ther. 2020;33(6):e14488. doi:10.1111/dth.14488. 33131170

[18] Haedersdal M , Sakamoto FH, Farinelli WA, Doukas AG, Tam J, Anderson RR. Fractional CO(2) laser-assisted drug delivery. Lasers Surg Med. 2010;42:113–22. 20166154 10.1002/lsm.20860

[19] Acosta S , Ureta E, Yañez R, Oliva N, Searle S, Guerra C. Effectiveness of intralesional triamcinolone in the treatment of keloids in children. Pediatr Dermatol. 2016;33:75–9. 26758090 10.1111/pde.12746

[20] Weshahy AH , Abdel Hay R. Intralesional cryosurgery and intralesional steroid injection: a good combination therapy for treatment of keloids and hypertrophic scars. Dermatol Ther. 2012;25:273–6. 22913446 10.1111/j.1529-8019.2012.01456.x

